# Interplay Between Receptor-Ligand Binding and Lipid Domain Formation Depends on the Mobility of Ligands in Cell-Substrate Adhesion

**DOI:** 10.3389/fmolb.2021.655662

**Published:** 2021-04-12

**Authors:** Long Li, Xiaohuan Wang, Helong Wu, Yingfeng Shao, Huaping Wu, Fan Song

**Affiliations:** ^1^ State Key Laboratory of Nonlinear Mechanics (LNM) and Beijing Key Laboratory of Engineered Construction and Mechanobiology, Institute of Mechanics, Chinese Academy of Sciences, Beijing, China; ^2^ School of Engineering Science, University of Chinese Academy of Sciences, Beijing, China; ^3^ College of Mechanical Engineering, Zhejiang University of Technology, Hangzhou, China

**Keywords:** cell adhesion, receptor-ligand binding, nanoscale lipid cluster, ligand mobility, phase separation, binding constant

## Abstract

Cell-cell adhesion and the adhesion of cells to extracellular matrix are mediated by the specific binding of receptors on the cell membrane to their cognate ligands on the opposing surface. The adhesion receptors can exhibit affinity for nanoscale lipid clusters that form in the cell membrane. Experimental studies of such adhesion systems often involve a cell adhering either to a solid surface with immobile ligands or a supported lipid bilayer with mobile ligands. A central question in these cell-substrate adhesions is how the mobility of the ligands physically affects their binding to the adhesion receptors and thereby the behavior of the nanoscale lipid clusters associated with the receptors. Using a statistical mechanical model and Monte Carlo simulations for the adhesion of cells to substrates with ligands, we find that, for mobile ligands, binding to adhesion receptors can promote the formation of mesoscale lipid domains, which in turn enhances the receptor-ligand binding. However, in the case of immobile ligands, the receptor-ligand binding and the tendency for the nanoscale lipid clusters to further coalesce depend on the distribution of the ligands on the substrate. Our findings help to explain why different adhesion experiments for identifying the interplay between receptor-ligand binding and heterogeneities in cell membranes led to contradictory results.

## 1 Introduction

Cell-cell adhesion and the adhesion of cells to the extracellular matrix (ECM), mediated by the specific binding of receptors and ligands anchored to the apposing surfaces, governs numerous biological processes such as signal transduction, immune responses, cell locomotion, tissue formation, as well as cancer invasion and metastasis (Weikl and Lipowsky, 2004; Krobath et al., 2009; van der Merwe and Dushek, 2011; Benham-Pyle et al., 2015; Chang et al., 2020; Li and Song, 2020; Romani et al., 2021). In cell-cell adhesion, both receptors and ligands are laterally mobile, whereas in cell-ECM adhesion, mobile receptors on the cell membranes often bind immobile ligands presented on the apposing surface (Sackmann and Smith, 2014). The key quantity to characterizing the receptor-ligand binding is the equilibrium constant K=[RL]/[R][L] (Bell, 1978; Li et al., 2019; Steinkühler et al., 2019; Zhu et al., 2019) that involves the area concentrations of receptor-ligand complexes [RL], unbound receptors [R] and unbound ligands [L]. A variety of techniques such as micropipette aspiration (Huang et al., 2010; Zhu et al., 2019), flow chamber (Limozin et al., 2016), atomic force microscopy (Doan et al., 2011), as well as fluorescence spectroscopy (O’Donoghue et al., 2013), have been used to directly measure the two-dimensional binding constant. In contrast to the binding of soluble receptors and ligands in aqueous solution, theoretical, simulation and experimental studies have revealed that the binding constant *K* of intercellular adhesion molecules not only depends on the specific interaction of receptors and ligands, but also on the membrane physical properties (Hu et al., 2013; Steinkühler et al., 2019).

The cell adhesion receptors can be associated with nanoscale lipid clusters in the cell membranes (Shao et al., 2015; Su et al., 2016; Stone et al., 2017). These clusters enriched in cholesterol and sphingolipids are specialized liquid-ordered membrane microdomains and termed as lipid rafts. They can be stabilized and made to coalesce, forming platforms that function in membrane signaling (Lingwood and Simons, 2010; Sezgin et al., 2017). Cell-cell adhesion experiments show that the adhesion of immune cells to the antigen presenting cells leads to the accumulation of lipid rafts and reorganization of signaling proteins at the immunological synapse, which in turn facilitate the receptor-ligand mediated antigen presentation and immune cell activation (Anderson and Roche, 2015). Disrupting the rafts in T cell membrane *via* cholesterol depletion directly reduces the binding constant *K* of the T cell receptor (TCR) and peptide-major histocompatibility complexes (pMHC) (Huang et al., 2010). Experiments of mimetic systems indicate that the adhesion of giant vesicles decorated with biotin to streptavidin-functionalized supported bilayers stabilizes raft heterogeneity in both isolated plasma membrane vesicles and synthetic vesicles, leading to protein aggregation within the adhesion zone. Destabilizing raft and protein heterogeneities in vesicles adversely affects the biotin-streptavidin mediated stable adhesion (Zhao et al., 2013). For the adhesion of cells to substrates with immobile ligands mimicking cell-ECM adhesion, experimental studies led to contradictory results regarding the interplay between receptor-ligand binding and coalescence of raft domains (Gaus et al., 2006; Mitchell et al., 2009; Evani and Ramasubramanian, 2016; Son et al., 2017). For example, Son et al. (Son et al., 2017) found that integrin-mediated adhesion of human aortic endothelial cells to a substrate facilitates raft domain formation and integrin clustering, which increase the cell-substrate adhesion. Conversely, Evani and Ramasubramanian (Evani and Ramasubramanian, 2016) observed that the adhesion of infected monocytes to the microchannels coated with E-selectin is enhanced due to the increased uniformity of lipid raft and CD44 distribution. These studies raise the question of how the mobility of the ligands physically affects their binding to the cell adhesion receptors and thereby the behavior of the nanoscale lipid clusters associated with the receptors.

**FIGURE 1:**
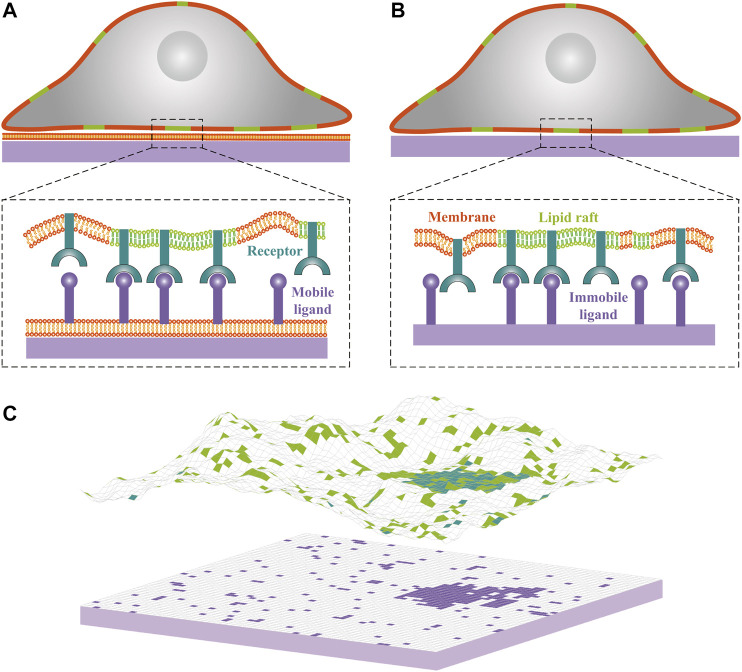
Cartoons of cell-substrate adhesion with **(A)** mobile ligands anchored to a lipid bilayer supported by a planar substrate and **(B)** immobile ligands directly attached to a planar substrate. Lipid rafts in the cell membrane are shown in green, cell adhesion receptors in blue, and ligands in purple. **(C)** Snapshot from Monte Carlo simulations of one membrane that adheres to a planar supported bilayer. Lipid rafts, receptors and ligands are indicated by square patches with the same color code as in panels **(A)** and **(B)**. Each receptor or ligand occupies a single patch. The adhesion receptors exhibit weak affinity for lipid rafts, as reflected by accumulation of the blue patches within the green patches. One receptor-ligand complex forms only if the receptor and ligand are opposite each other and if the distance between receptor and ligand patches is within receptor-ligand binding range. Lipid rafts have a tendency to coalesce partially because of their hydrophobic mismatch with the membrane matrix.

In this paper, we use methods of classical statistical mechanics and Monte Carlo (MC) simulations to study the interplay between receptor-ligand binding and raft domain formation for two typical experimental systems of cell adhesion, where multicomponent membranes adhere to a supported planar bilayer with mobile ligands, or to a planar substrate with immobile ligands, as illustrated in Figure 1. We find that, in a biologically relevant range of model parameters, the interplay between receptor-ligand binding and raft domain formation depends strongly on the ligand mobility. In the former adhesion system with mobile ligands, the receptor-ligand binding can enhance the coalescence of the rafts associated with the receptors by means of membrane-mediated attraction between the receptor-ligand complexes. The raft coalescence in turn facilitates the formation of additional receptor-ligand complexes due to the less loss in the configurational entropy of the membrane. This suggests that the receptor-ligand binding and raft domain formation in cell-supported bilayer adhesion system are mutually beneficial. In the latter adhesion system with immobile ligands, the interplay depends sensitively on the distribution of the ligands immobilized on the substrate. Our results show that for ligands uniformly or randomly immobilized on the substrate, the receptor-ligand binding disfavors the aggregation of rafts, whereas for ligands immobilized in the form of clusters on the substrate, receptor-ligand binding and raft aggregation can cooperate.

## 2 Model and Methods

We employ a statistical-mechanical model that has been widely used to study both the dynamics (Bahrami and Weikl, 2018; Knezevic et al., 2018) and equilibrium behavior (Weikl et al., 2016; Li et al., 2018b) of cell adhesion. In our model of cell-substrate adhesion, the cell membrane, supported bilayer, as well as rigid planar substrate are represented by two-dimensional square lattices of size *a*, as shown in Figure 1C. The system bending energy can be written in a discretized form as (Li et al., 2020; Li et al., 2018a)

$${ℋ}_{\text{me}}=\frac{\kappa }{2a^{2}}\sum_{i}{\left(\Delta_{d}l_{i}\right)}^{2},$$ (1)

which governs the elastic deformation of the cell membrane. Here *κ* is the bending rigidity of cell membrane, $l_{i}$ is the local separation between two apposing patches with index i, and $\Delta_{d}l_{i}$ is the discretized Laplacian of the separation field $\left\{l_{i}\right\}$.

We set the lattice size a=10 nm to match the exclusion radius of adhesion proteins (Tsourkas et al., 2008). Then, by analogy to lattice-gas-type models, a single square patch can accommodate only one receptor or one ligand. The spatial distribution of adhesion proteins is described by the composition field $\left\{m_{i}^{o}\right\}$ with values $m_{i}^{\text{o}}=0\;\text{or}\;1$ indicating the absence or presence of adhesion protein at patch i. The superscript o=+,− distinguishes the upper cell membrane and lower supported bilayer or substrate. Unlike the cell-supported bilayer system with mobile receptor and ligand proteins, the ligands are immobilized on the substrate for cell-substrate adhesion without lipid bilayer. One receptor-ligand complex forms only if receptor and ligand are located at opposite membrane patches with a local separation $l_{i}$ within the binding range, i.e., $l_{c}-l_{b}/2<l_{i}<l_{c}+l_{b}/2$, where $l_{c}$ and $l_{b}$ denote the length of receptor-ligand complex and the width of the binding potential, respectively. The adhesion energy from the specific receptor-ligand binding is (Krobath et al., 2009; Rozycki et al., 2010)

$${ℋ}_{R-L}=\sum_{i}V_{b}m_{i}^{+}m_{i}^{-}=-u_{b}\sum_{i}m_{i}^{+}m_{i}^{-}\theta \left(\frac{l_{b}}{2}-\left|l_{i}-l_{c}\right|\right),$$ (2)

where the square-well binding potential $V_{b}$ has the depth $u_{b}$, θ(⋯) denotes the Heaviside’s step function. The potential in Eq. 2 effectively takes into account the binding specificity of rigid protein. For the case of flexible proteins, their conformations might play a role in the binding, which requires more detailed modeling, e.g., the bead-spring model of polymer chains. The conformational flexibility of adhesion proteins shall be investigated in future studies.

In our model, only the cell membrane contains lipid raft. Each raft patch is represented by a square lattice of size a×a. The spatial distribution of lipid rafts is then described by the composition fields $\left\{n_{i}^{+}\right\}$ with values 0 or 1 indicating whether a lipid raft is absent or present in lattice site *i* of cell membrane. To capture the protein affinity for lipid raft, we introduce the energy gain $u_{\text{a}}$ for a protein to move from a raft domain to the membrane matrix. The total energy contributed by the coupling between receptor molecules and lipid rafts is (Li et al., 2017a)

$${ℋ}_{r-p}=-u_{a}\underset{i}{\sum }\left(n_{i}^{+}m_{i}^{+}\right).$$ (3)

To consider the tendency for lipid rafts to coalesce due to their hydrophobic mismatch with the membrane matrix, we assume a contact energy *u* for nearest-neighbor raft patches. The total raft-raft contact energy is then described by (Li et al., 2017b)

$${ℋ}_{r-r}=-u\sum_{\langle i,j\rangle }\left(n_{i}^{+}n_{j}^{+}\right).$$ (4)

which sums over all pairs of nearest-neighbor raft patches *i* and *j*.

We employ the Metropolis MC method to simulate the adhesion systems with total energy ${ℋ}_{ad}={ℋ}_{me}+{ℋ}_{R-L}+{ℋ}_{r-p}+{ℋ}_{r-r}$. There are three types of MC trial moves including lateral translations of proteins and lipid rafts modeled as a hopping process to mimic their diffusion, and vertical displacements of cell membrane patches to simulate membrane shape fluctuations. Specifically, mobile proteins and raft patches are attempted to move separately to one of the four neighboring lattice sites in the trial moves. In the trial moves of raft sites, the receptors within the raft sites do not move along with the rafts. The proportion of these trial moves during one MC cycle is chosen based on the physical time scales of the three motions, as done in our earlier work (Li et al., 2017a). The three types of MC trial moves lead to possible variations in conformational energy ${ℋ}_{ad}$ by changing the composition fields $\left\{m_{i}^{+}\right\}$, $\left\{m_{i}^{-}\right\}$, and $\left\{n_{i}^{+}\right\}$, and local separation field $\left\{l_{i}\right\}$ between two apposing patches. According to the standard Metropolis algorithm, these trial moves will be always accepted if the resulted change in conformational energy $\Delta {ℋ}_{ad}<0$, otherwise accepted with a ratio exp $\left(-\text{Δ}{ℋ}_{\text{ad}}/k_{\text{B}}T\right)$ with $k_{\text{B}}$ the Boltzmann constant and T the absolute temperature. In addition, to avoid the overlap between the upper cell membrane and the lower supported bilayer or substrate, all trial vertical moves of cell membrane patches leading to $l_{i}<0$ are rejected. Assuming the typical diffusion coefficient $D=1\;{\text{μm}}^{2}/\text{s}$ (Gambin et al., 2006; Ramadurai et al., 2009) for membrane proteins, the time $t=a^{2}/4D$ for the proteins traveling a distance of a=10 nm to a neighboring lattice site is of the order of 10 µs. Thus, a MC cycle corresponds to a physical time of 10 µs. We identify phase transitions in the membrane system by monitoring the heat capacity per lattice site, $C_{V}=\left(\langle {ℋ}_{ad}^{2}\rangle -{\langle {ℋ}_{ad}\rangle }^{2}\right)/\left(Nk_{B}T^{2}\right)$ as a function of the model parameters. Here, 〈…〉 denotes the ensemble average, and *N* is the total number of discretized membrane patches.

We have simulated the adhering membrane with an area of $A_{me}=600\times 600\;{\text{nm}}^{2}$ under periodic boundary conditions. In each MC simulation, the receptors, raft patches, and mobile ligands are all randomly distributed in the apposing surfaces at the beginning of the simulation unless otherwise specified. Note that the initial distributions of proteins and lipid rafts, however, do not affect the binding constant and phase behavior, since the equilibrium quantities do not depend on the dynamic properties of the system. In each simulation, we perform a relaxation run of $5\times 10^{7}$ MC cycles for thermal equilibration and a subsequent run of $5\times 10^{7}$ MC cycles for statistical sampling. We then compute the area concentrations of the unbound receptors, the unbound ligands and the bound receptor-ligand complexes to calculate the receptor-ligand binding constant K=[RL]/([R][L]). The MC data points are statistical averages of 10 independent runs, and the error bars represent the standard deviation (SD) of the 10 measurements. We set the simulation parameters according to existing literature data. Specifically, we choose the bending rigidity $\kappa =10\;k_{B}T$ for fluctuating cell membrane (Xu et al., 2015). To characterize the square-well potential, we take binding energy $u_{b}=4\;k_{B}T$ or $6\;k_{B}T$, potential range $l_{b}=1$ nm, and receptor-ligand complex length $l_{c}=15$ nm (Krobath et al., 2009; Rozycki et al., 2010). The coupling energy between receptor molecules and lipid rafts $u_{a}=3\;k_{B}T$ is adopted so that the protein concentration in the raft domains is within the experimentally measured range of around $10^{3}-10^{4}$ µm^−2^ (Simons and Toomre, 2000). In our simulations, we vary the area concentration of receptor molecules up to 2000 µm^−2^ (Hu et al., 2013), and change the area fraction of the raft domains *x* up to 30% of the membrane surface area (Simons and Toomre, 2000; Foster et al., 2003; Fallahi-Sichani and Linderman, 2009).

We also develop a mean field (MF) theory to study the phase behavior of adhesion system with planar supported membrane containing mobile ligand molecules, as detailed in the Supporting Information (SI). Briefly, we first introduce the grand-canonical Hamiltonian, as given by Supplementary Eq. S1 in SI. Then we treat the raft-raft interaction in a MF manner and derive the approximate free energy of the system by considering the contact probability $P_{b}$, which is the equilibrium fraction of bound membrane patches in the reference system of two homogeneous membranes and can be determined by MC simulation. Finally, by solving the self-consistent equations of the system (Supplementary Eq. S9), we can identify the phase transition points.

## 3 Results

### 3.1 Adhesion of Cell Membrane to the Planar Supported Membrane with Mobile Ligands

Before exploring the interplay between receptor-ligand binding and raft domain formation, we first consider a limiting case of rigid and planar membranes ($\kappa_{r}=\kappa_{m}=\infty$) without receptor-ligand binding. In this limit, the adhesion system reduces to the two-dimensional lattice gas, and its phase behavior can be described by the exact solution of the Ising model on two-dimensional square lattice (Montroll et al., 1963). At small values of raft-raft contact energy *u*, the membrane is in a homogenous state with lipid rafts distributed more or less uniformly within the cell membrane. At large values of u the rafts can coalesce into mesoscopic domain and the system undergoes phase separation. The transition between the homogeneous and phase-separated state occurs for $u>u_{0}^{*}$, where the critical raft-raft contact energy $u_{0}^{*}=2\ln\left(1+\sqrt{2}\right)k_{B}T$. Within the MF theory, however, $u_{0}^{*}=k_{B}T$.

**FIGURE 2:**
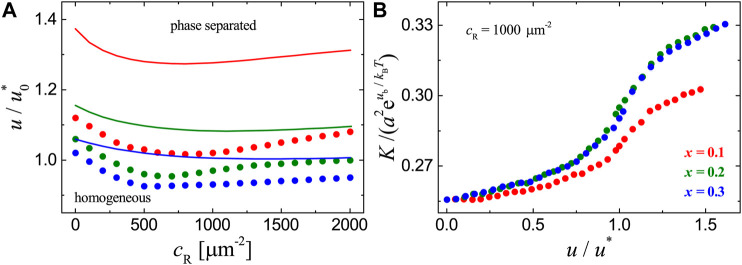
Results for the adhesion systems with mobile ligands and receptor-ligand binding strength $u_{b}=6k_{B}T$, raft-receptor affinity $u_{a}=3k_{B}T$, raft area fraction x=0.1,0.2 and 0.3, total concentration of ligands $c_{L}=1000\;\mu m^{-2}$. **(A)** Phase diagrams. The dots are phase transition points determined from the $C_{\text{V}}$ versusu plots in MC simulations as explained in the main text. The lines show the location of phase transition obtained from MF calculation. $u_{0}^{\text{*}}$ is the raft-raft contact energy at the critical point in the absence of receptors (receptor concentration $c_{R}=0$), i.e., without receptor-ligand binding. The raft-raft contact energy at the critical point $u_{0}^{*}=2\ln\left(1+\sqrt{2}\right)k_{B}T$ for MC data and $u_{0}^{*}=k_{B}T$ for MF calculations. **(B)** Binding constant *K* vs. raft-raft contact energy *u* at $c_{L}=100\;\mu m^{-2}$. $u^{\text{*}}$ is the raft-raft contact energy at the corresponding phase transition point of each system.

Consider now the adhesion systems with mobile receptors that have affinity for lipid rafts in the cell membrane and mobile ligands anchored to a planar supported bilayer as illustrated in Figure 1A. To first explore the effect of the receptor-ligand binding on the phase behavior, we perform a series of MC simulations with the receptor concentration $c_{\text{R}}$ ranging from 0 to 2000 µm^−2^, raft-raft contact energy *u* in the range from $0.8u_{0}^{*}$ to $1.2u_{0}^{*}$, and raft area fraction x=0.1,0.2, or 0.3. The phase diagrams in Figure 2A from MC simulations (dots) show that receptor-ligand binding reduces the value of *u* at which the phase separation occurs. This is also qualitatively confirmed by the MF calculations as shown in Figure 2A (lines). The quantitative discrepancies between the MC and MF results is due to the fact that fluctuations in the local concentration of rafts are neglected within MF theory. The effect of receptor-ligand binding on the phase separation is related to the thermally-excited shape fluctuations of the cell membrane and can be explained as follows: the receptor-ligand complexes locally clamp the two apposing surfaces and suppress the cell membrane fluctuations. This gives rise to an effective attraction between receptor-ligand complexes, since the cell membrane can adopt more configurations when the complexes are in close proximity than far apart (Krobath et al., 2009; Speck et al., 2010; Fenz et al., 2017). This membrane-medated lateral attraction between the receptor-ligand complexes enhances the coalescence of lipid rafts that are associated with the receptors. Therefore, the transition from homogeneous to phase-separated states in the adhesion systems can take place at smaller contact energies *u*. In addition, we note that the value of *u* at which the phase separation occurs first decreases and then increases with the concentration $c_{R}$ of receptors. This can be understood from the membrane-mediated, fluctuation-induced attraction between receptor-ligand complexes. At small values of $c_{R}$, this attraction facilitates the clustering of lipid rafts that are associated with the bound receptors. At large values of $c_{R}$, the formation of more receptor-ligand complexes largely suppresses the membrane shape fluctuations and therefore weakens the attraction, leading to the increase of *u* for phase separation to occur.

To clearly show how the raft domain formation affects the receptor-ligand binding, we plot the binding constant *K* measured in MC simulations as a function of raft-raft contact energy *u* in Figure 2B. For each adhesion system with fixed concentration $c_{\text{R}}$ of receptors and area fraction *x* of rafts, *K* increases with *u* and there appears a jump in *K* as the system approaches the phase transition point ($u/u^{*}=1$). Increasing raft-raft contact energy *u* leads to the aggregation of raft-associated receptors and therefore enhances the binding, because the receptor-ligand complexes formed within raft domains will cost less configurational entropy of the cell membrane. Our results in Figure 2 indicate that the receptor-ligand binding facilitates raft domain formation in the cell-substrate adhesion systems with mobile ligands, and vice versa.

### 3.2 Adhesion of Cell Membrane to the Substrate with Immobile Ligands

**FIGURE 3:**
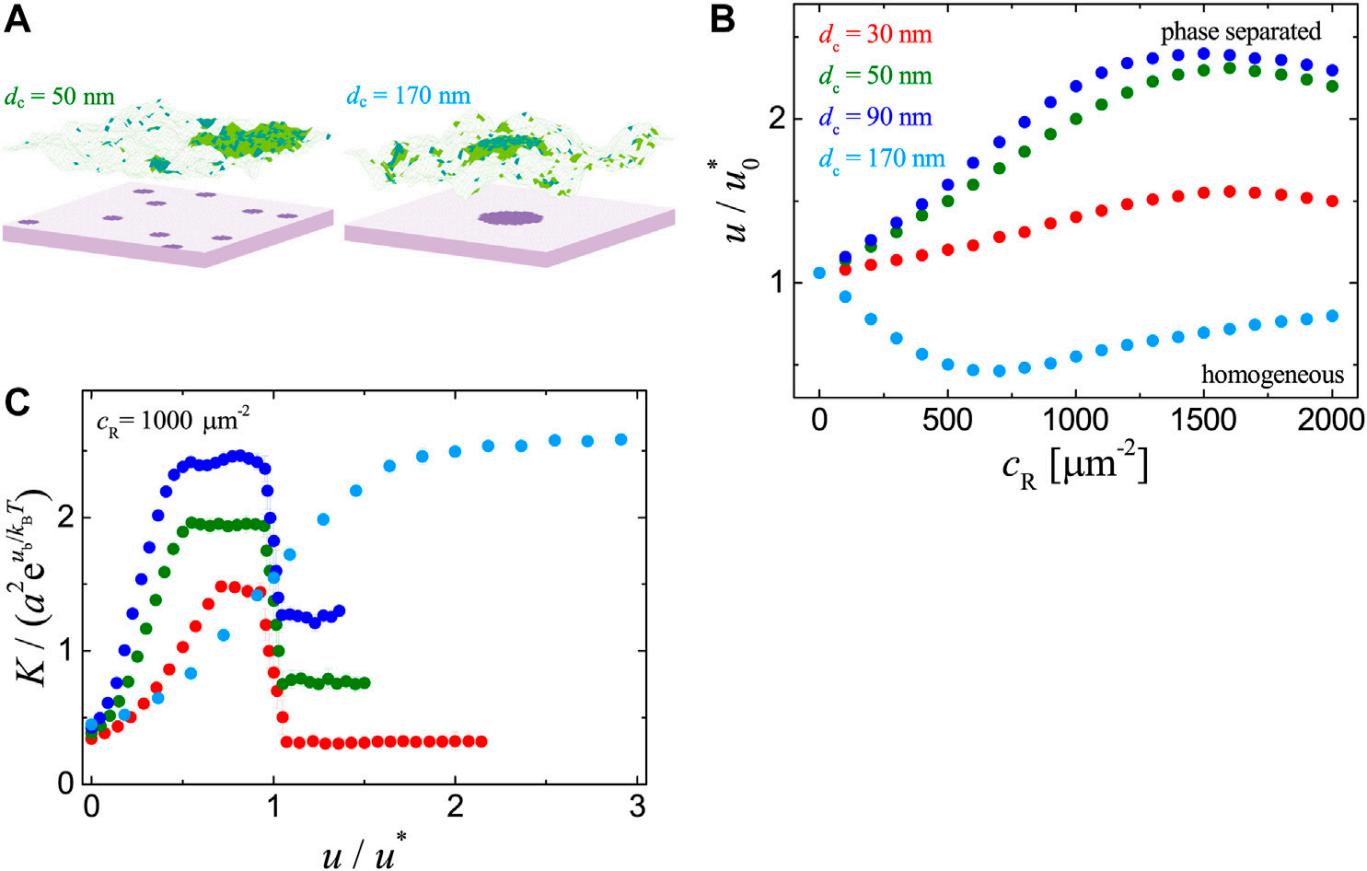
Results for the adhesion systems with immobile ligands in clusters as obtained from MC simulations with $u_{b}=4k_{B}T$, $u_{a}=3k_{B}T$, x=0.2, $c_{L}\approx 580\;{\text{μm}}^{-2}$. **(A)** Simulation snapshots with 10 **(left)** and 1 **(right)** clusters of ligands. The diameter of each cluster is $d_{c}=$ 50 nm and 170 nm, respectively. **(B)** phase diagrams for systems with ligand clusters of different diameters as specified in the legend. **(C)** Binding constant *K* vs. raft-raft contact energy *u* for $c_{R}=1000\;{\text{μm}}^{-2}$. The symbols have the same physical meanings as in Figure 2.

We now turn to the cell-substrate adhesion systems with ligands immobilized on the flat substrate as illustrated in Figure 1B. We consider three types of distributions for the immobile ligands, namely, (i) in clusters, (ii) uniform, and (iii) random. For the first type of ligand distribution, we perform MC simulations with raft area fraction x=0.2 and ligands in one or multiple clusters of nearly circular shape at the total concentration $c_{L}\approx 580$ µm^−2^ as shown in Figure 3A. The ligand clusters are immobilized and randomly distributed on the substrate. Figure 3B shows the phase diagrams for different diameters $d_{c}$ of the ligand clusters as obtained from statistical average over 10 independent runs. In the case of multiple clusters (*d*_c_ = 30, 50, and 90 nm), the raft-raft contact energy *u* for the adhesion system to phase separation is increased by the presence of receptors, i.e., the binding of receptors to ligands immobilized in multiple clusters hinders the formation of large raft domains. This is due to the fact that the coalescence of small raft domains needs to release the receptors that are associated with the rafts and already bound to immobile ligands (energy cost of $u_{a}$ per receptor), or to break the receptor-ligand bonds (energy cost of $u_{b}$ per bond). Note that this result is contrary to the previous cell-supported bilayer adhesion systems with mobile ligands in which the receptor-ligand binding promotes the phase separation; see Figure 2A. However, in the case of one single cluster ($d_{\text{c}}=170\;\text{nm}$), the raft-raft contact energy *u* at which phase transition occurs is decreased in the presence of receptors, since the receptor-ligand binding here enhances the clustering of receptors and therefore facilitates the coalescence of rafts associated with the receptors.

We further analyze the dependence of receptor-ligand binding constant *K* on the raft-raft contact energy *u*. As illustrated in Figure 3C, in the case of one single ligand cluster ($d_{c}=170\;\text{nm}$), K increases with *u* and saturates for large *u*. The clustering of raft-associated receptors induced by raft coalescence enhances their binding to the ligands immobilized in the single cluster, originating from the translational entropy of rafts (Li et al., 2018a) and configurational entropy of the membrane. The physical picture is that, a raft domain with one of its associated receptors bound to an immobile ligand on the opposing surface will lose less translational entropy upon the formation of another complex if the two complexes stay close (Li et al., 2018a). The flexible membrane can also take more configurations when the receptor-ligand complexes are nearby. At large *u*, a mesoscale raft domain forms within the membrane and it contains most of the receptors that are able to bind with the immobile ligands, leading to a plateau for *K*. In the case of multiple ligand clusters (*d*_c_ = 30, 50, and 90 nm), *K* first increases with *u* and reaches a plateau, then decreases abruptly in the vicinity of phase transition point, and eventually levels off for *u* much larger than $u^{\text{*}}$. Such complex dependence can also be understood by considering the raft-induced receptor aggregation. At small *u*, there exists many separate and small raft domains, some of which have no opposing ligand clusters and the associated receptors thus have no chance to bind ligands. As *u* increases, the rafts coalesce into less but large domains, and the percentage of those excessive receptors decreases, leading to an increase in *K*. Once each of the multiple ligand clusters is linked to a large raft domain *via* receptor-ligand complexes, *K* remains unchanged with *u* until the system is brought close to the transition point (i.e., $u/u^{\text{*}}∼1$), at which the raft domains tend to merge to one and *K* decreases rapidly since there will be more unbound receptors associated with the one single raft domain of size larger than the apposing ligand clusters. The abrupt change in *K* suggests that *K* is an indicator of phase separation for the cell-substrate adhesion systems with multiple immobile ligand clusters.

**FIGURE 4:**
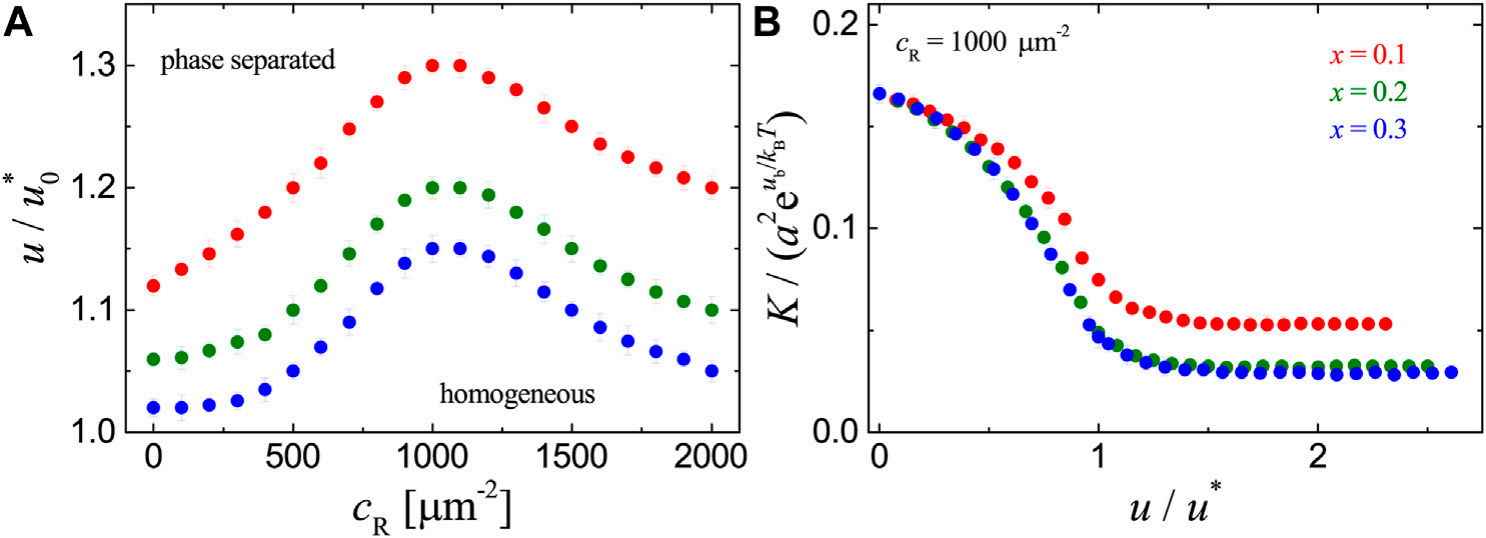
Results for the adhesion systems with ligands immobilized uniformly on the substrate as obtained from MC simulations with $u_{b}=6k_{B}T$, $u_{a}=3k_{B}T$, $c_{L}=625\;{\text{μm}}^{-2}$, x=0.1,0.2 and 0.3. **(A)** phase diagrams. **(B)** Binding constant *K* vs. the raft contact energy *u* at $c_{R}=1000\;{\text{μm}}^{-2}$. The symbols have the same physical meanings as in Figure 2.

**FIGURE 5:**
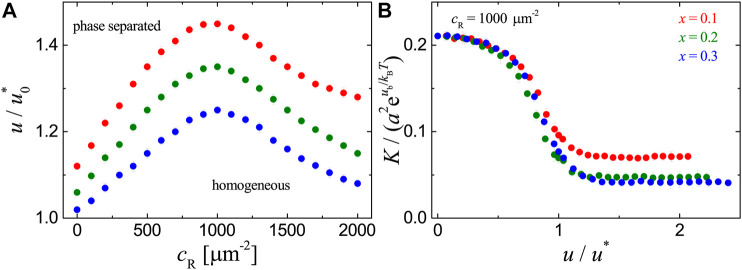
Results for the adhesion systems with ligands immobilized randomly on the substrate as obtained from MC simulations with $u_{b}=6k_{B}T$, $u_{a}=3k_{B}T$, $c_{L}=625\;{\text{μm}}^{-2}$, x=0.1,0.2 and 0.3. **(A)** phase diagrams. **(B)** Binding constant *K* vs. raft-raft contact energy *u* at $c_{R}=1000\;{\text{μm}}^{-2}$. The symbols have the same physical meanings as in Figure 2.

We then consider the adhesion systems with ligands uniformly or randomly immobilized on the substrate. We’ve performed MC simulations for the two cases with ligand concentration $c_{L}=625\;{\text{μm}}^{-2}$ and raft area fraction x=0.1, 0.2, and 0.3. Figure 4A shows that, for uniformly-distributed ligands, the raft-raft contact energy *u* at the phase transition point increases with receptor concentration $c_{\text{R}}$ and reaches a maximum at $c_{R}\approx 1000{\text{ μm}}^{-2}$. So the binding of receptors to ligands that are uniformly immobilized on the substrate disfavors phase separation, since large raft domains form within the membrane at the expense of weakening receptor-raft coupling and breaking receptor-ligand bonds. Figure 4B shows that at fixed $c_{R}$, the receptor-ligand binding constant *K* decreases with the raft-raft contact energy *u* and starts to levels off as *u* becomes even larger than the value $u^{\text{*}}$ at the phase transition point. The decrease in *K* is caused by the energetic penalty paid for the receptors within lipid-raft domains in order to bind to substrate-immobilized ligands outside of the region apposing the raft domains. The results for randomly distributed ligands in Figure 5 are obtained from statistical average over 10 independent realizations, and look similar to those in Figure 4. A careful comparison of Figures 4, 5 reveals that the phase separation occurs at a slightly larger value of raft-raft contact energy *u* and the receptor-ligand binding constant *K* is greater for randomly-distributed ligands than for uniformly-distributed ligands, given the other parameters are the same in both cases. The difference in *K* can be attributed to local aggregation of the randomly-distributed ligands that costs less loss in both translational entropy of the rafts and conformational entropy of the membrane upon receptor-ligand binding. Once the receptors bind strongly to randomly immobilized ligands, the coalescence of rafts associated with those receptors becomes expensive. Figures 4, 5 indicate that the receptor-ligand binding and phase separation negatively affect each other for adhesion systems with ligands uniformly or randomly immobilized on the substrate.

As mentioned in Introduction, there is a discrepancy regarding the interplay between receptor-ligand binding and lipid raft coalescence in adhesion experiments with mobile (Huang et al., 2010; Anderson and Roche, 2015) and immobile ligands (Gaus et al., 2006; Mitchell et al., 2009; Evani and Ramasubramanian, 2016; Son et al., 2017). Our results suggest that the discrepancy may be caused by the mobility of ligands in cell-substrate adhesion. Consistent with the experiments with mobile ligands, we find that the receptor-ligand binding and raft domain formation are mutually beneficial. For cell-substrate adhesion with immobile ligands, we find that the distribution of ligands immobilized on the substrate is an important factor. Depending on the ligand distribution, the receptor-ligand binding and raft coalescence can be mutually beneficial or not. Experimentally, the immobilization of ligand proteins on the substrate depends on temperature, pH, buffer composition, ionic strength, as well as the properties of protein and substrate (Rabe et al., 2011). Different conditions can lead to different distributions of ligands on the substrate, which affects the interplay between receptor-ligand binding and raft domain formation.

## 4 Conclusion

We’ve investigated cell-substrate adhesion mediated by the binding of cell adhesion receptors to ligands that are either anchored to a substrate-supported planar lipid bilayer or directly immobilized on the flat substrate by means of MC simulations and MF calculations based on a classical statistical mechanical model. The model takes into account shape fluctuations of the cell membrane and weak coupling of adhesion receptors to lipid rafts within the cell membrane. We focused on how the ligand mobility plays a role in the interplay between the receptor-ligand binding and raft domain formation. In the biologically relevant range of model parameters, we find that, for the adhesion systems with mobile ligands, the receptor-ligand binding promotes the coalescence of raft domains, which in turn enhances the binding. The lateral attraction between receptor-ligand complexes induced by the membrane shape fluctuations is responsible for the positive correlation.

In the adhesion systems with immobile ligands, we find that the interplay between receptor-ligand binding and raft domain formation depends on the distribution of ligands. For uniformly or randomly immobilized ligands, their binding to raft-associated receptors disfavors the aggregation of rafts and vice versa. For substrate-immobilized ligands in clusters, the receptor-ligand binding and raft domain formation can be mutually beneficial or not depending on whether there exists one single or multiple clusters of immobile ligands. The interplay can be understood by considering the loss in membrane configurational entropy and the raft translational entropy upon binding. Our findings not only reveal that ligand mobility and distribution are important physical factors in cell-substrate adhesion, but also help to explain the seemingly contradictory results regarding the interplay between R-L binding and raft heterogeneities in cell-substrate adhesion experiments (Gaus et al., 2006; Mitchell et al., 2009; Huang et al., 2010; Anderson and Roche, 2015; Evani and Ramasubramanian, 2016; Son et al., 2017), where the mobility and distribution of ligands on the substrate should be carefully taken into account.

## Data Availability

The original contributions presented in the study are included in the article/Supplementary Material, further inquiries can be directed to the corresponding authors.
