# Supplementary material for: Interplay Between Receptor-Ligand Binding and Lipid Domain Formation Depends on the Mobility of Ligands in Cell-Substrate Adhesion
Source: Front Mol Biosci. 2021 Apr 12;8:655662. doi: 10.3389/fmolb.2021.655662 (PMC8112205; doi:10.3389/fmolb.2021.655662)
Supplement: Supplementary file 1 [file datasheet1.pdf]

Supplementary Material

1 MEAN FIELD THEORY

We implement the mean field (MF) theory for adhesion system, wherein cell membrane adheres to planar supported bilayer containing mobile ligand molecules. We start with the grand-canonical ensemble in which the concentrations of adhesion proteins and lipid rafts are determined by the chemical potentials $\mu_{\rm p}$ and $\mu_{\rm r}$, respectively. The grand-canonical Hamiltonian is then given by

$$\mathcal{H} = \mathcal{H}_{\rm ad} - \sum_i \mu_{\rm r}^+ n_i^+ - \sum_i \left(\mu_{\rm p}^+ m_i^+ + \mu_{\rm p}^- m_i^-\right) \tag{S1}$$

where $\mathcal{H}_{\rm ad}$ is the total energy of canonical ensemble as given in main text. The superscript $+,-$ distinguishes the upper cell membrane and lower supported bilayer. Transforming raft variables $n_i^+ = 0, 1$ to spin variables $s_i^+ = 2n_i^+ - 1 = \pm 1$, and using MF approximation $s_i^+ s_j^+ \approx \langle s_i^+ \rangle s_i^+ + \langle s_j^+ \rangle s_j^+ - \langle s_i^+ \rangle \langle s_j^+ \rangle$ with the average $s = \langle s_i^+ \rangle = \langle s_j^+ \rangle$, we obtain MF Hamiltonian

$$\begin{aligned} \mathcal{H}_{\rm MF} &= \mathcal{H}_{\rm me} + \mathcal{H}_{\rm R-L} + \\ & \sum_i \left[\epsilon_{\rm r}^+ - U_{\rm effr} s_i^+ - \frac{u_{\rm a}}{2}\left(s_i^+ + 1\right) m_i^+ - \mu_{\rm p}^+ m_i^+ - \mu_{\rm p}^- m_i^- + V_{\rm b} m_i^+ m_i^-\right] \end{aligned} \tag{S2}$$

with $\epsilon_{\rm r}^+ = \frac{1}{2}\left[u(s^2-1) - \mu_{\rm r}^+\right]$, $U_{\rm effr} = u(1+s) + \frac{1}{2}\mu_{\rm r}^+$. Then the grand-canonical partition function is obtained as

$$\begin{aligned} \mathcal{Z}_{\rm MF} &= \left[\prod_i \int_0^\infty {\rm d}l_i\right] \left[\prod_i \sum_{s_i^+=\pm 1} \sum_{m_i^-=0,1} \sum_{m_i^+=0,1}\right] e^{-\beta \mathcal{H}_{\rm MF}} \\ &= e^{-N\beta\epsilon_{\rm r}^+} \left[\prod_i \int_0^\infty {\rm d}l_i\right] \left[e^{-\beta\mathcal{H}_{\rm me}} \prod_i \sum_{\sigma^+=\pm 1} w_{\sigma^+}(l_i)\right] \end{aligned} \tag{S3}$$

with $\beta = (k_{\rm B}T)^{-1}$ and

$$\begin{aligned} w_{\sigma^+}(l_i) &= \Big[e^{\beta U_{\rm effr}\sigma^+} + e^{\beta\left(U_{\rm effr}\sigma^+ + \mu_{\rm p}^-\right)} \\ & + e^{\beta\left(U_{\rm effr}\sigma^+ + \frac{1}{2}u_{\rm a}(\sigma^+ + 1) + \mu_{\rm p}^+\right)} + e^{\beta\left(U_{\rm effr}\sigma^+ + \frac{1}{2}u_{\rm a}(\sigma^+ + 1) + \mu_{\rm p}^+ + \mu_{\rm p}^- + u_{\rm b}\theta(l_{\rm b}/2 - |l_i - l_{\rm c}|)\right)}\Big] \end{aligned} \tag{S4}$$

By defining $A_{\sigma^+} = w_{\sigma^+}(l_i)|_{\theta(l_{\rm b}/2-|l_i-l_{\rm c}|)=0}$ and $B_{\sigma^+} = w_{\sigma^+}(l_i)|_{\theta(l_{\rm b}/2-|l_i-l_{\rm c}|)=1}$, the partition function given by Eq. (S3) can be rewritten as

$$\mathcal{Z}_{\rm MF} = e^{-N\beta\epsilon_{\rm r}^+}\left[\sum_{\sigma^+=\pm 1} A_{\sigma^+}\right]^N \left[\prod_i \int_0^\infty {\rm d}l_i\right] e^{-\beta\left[\mathcal{H}_{\rm me} + \sum_i V_{\rm b,eff}(l_i)\right]} \tag{S5}$$

where the effective binding potential $V_{\mathrm{b,eff}}(l_i) = -U_{\mathrm{b,eff}}\theta(l_\mathrm{b}/2 - |l_i - l_\mathrm{c}|)$ is a square-well potential with the same width l_b and location l_c as the R-L binding potential in Eq. (2) in the main text. The effective binding strength takes the form

$$U_{\mathrm{b,eff}} = k_\mathrm{B}T \ln \frac{\sum_{\sigma^+} B_{\sigma^+}}{\sum_{\sigma^+} A_{\sigma^+}} \tag{S6}$$

which is a function of parameters u_b, u_a, u, μ_p, μ_r and T. The free energy density is determined as

$$\mathcal{F} = -\frac{k_\mathrm{B}T}{N} \ln \mathcal{Z}_{\mathrm{MF}} = \epsilon_\mathrm{r}^+ - k_\mathrm{B}T \ln\Big[\sum_{\sigma^+=\pm1} A_{\sigma^+}\Big] + \mathcal{F}_0 \tag{S7}$$

where

$$\mathcal{F}_0 = -\frac{k_\mathrm{B}T}{N} \ln \left\{ \Big[\prod_i \int_0^\infty \mathrm{d}l_i\Big] e^{-\beta\left[\mathcal{H}_{\mathrm{me}} + \sum_i V_{\mathrm{b,eff}}(l_i)\right]} \right\} \tag{S8}$$

is the free energy density for two *homogeneous* membranes with Hamiltonian $\mathcal{H}_0 = \mathcal{H}_{\mathrm{me}} + \sum_i V_{\mathrm{b,eff}}(l_i)$.

Phase separation occurs if the free energy density $\mathcal{F}$ shows two equal minima separated by a maximum, implying that the first-order partial derivative $\partial\mathcal{F}/\partial s = 0$ has three roots s_1, s_2, and s_3, and the second-order partial derivative $\partial^2\mathcal{F}/\partial s^2$ is negative for one of the roots and positive for the other two. We can then obtain the self-consistent equation using the condition $\partial\mathcal{F}/\partial s = 0$

$$s = P_\mathrm{b} \frac{\sum_{\sigma^+} \sigma^+ B_{\sigma^+}}{\sum_{\sigma^+} B_{\sigma^+}} + (1 - P_\mathrm{b}) \frac{\sum_{\sigma^+} \sigma^+ A_{\sigma^+}}{\sum_{\sigma^+} A_{\sigma^+}} \tag{S9}$$

where $P_\mathrm{b} = -\partial\mathcal{F}_0/\partial U_{\mathrm{b,eff}} = \langle\theta(l_\mathrm{b}/2 - |l_i - l_\mathrm{c}|)\rangle_{\mathcal{H}_0}$ is the equilibrium fraction of bound membrane patches with $l_\mathrm{c} - l_\mathrm{b}/2 < l_i < l_\mathrm{c} + l_\mathrm{b}/2$ in the reference system, i.e., the so-called contact probability. P_b is determined by MC simulations for homogeneous membrane system with Hamiltonian $\mathcal{H}_0$. We then identify the phase transition points by numerically solving Eq. (S9) under the condition $\mathcal{F}(s_1) = \mathcal{F}(s_3)$.
